# Determinants of workforce availability and performance of specialists and general duty medical officers in Rajasthan, India

**DOI:** 10.1186/1753-6561-6-S5-O3

**Published:** 2012-09-28

**Authors:** Manish Tiwari, Sonu Pareek

**Affiliations:** 1 Shiv Charan Mathur Social Policy Research Institute, Rajasthan, India

## Introduction

The availability of adequate number of health professionals to manage health programmes alone may not necessarily lead to their successful implementation. The competencies and commitment of these professionals also need to be ensured. One of the biggest challenges, in this regard, has been poor capacity building at every rung of health care. The situation is further worsened by a shortfall in the human resource, especially specialists. There has often been a mismatch between the number of required and sanctioned posts and also between sanctioned and actual number of medical-officers and specialists posted in a health facility.

## Methods

In order to study and understand the issues of optimal workforce management and human resource development, a study on determinants of workforce availability and performance of specialists and general duty medical officers was conducted in Rajasthan. The study was done in association with the National Health Systems Resource Centre. The study aimed at assessing the gaps between the services expected and those provided at a facility level, along with analyzing the recruitment, compensation, transfer and training policy of medical officers and specialists.

On the basis of discussions with the National Health System Resource Centre and the State Health Department, four districts were selected on the basis of the Human Development Index (HDI). The institutions surveyed were District Hospital, Sub-Divisional Hospital, Community Health Centres and Primary Health Centres. In addition to an institutional survey, 40 doctors (30 general duty medical officers and 10 specialists) were also interviewed on a pre-designed format. The findings were validated through discussions with the state government.

## Results

It was found that one-fifth of the sample doctors had completed 25 years of service. Of all the doctors in the sample, 18% were lady doctors. A large number of doctors employed in the public health system hailed from rural background and expressed an inclination towards receiving specialized training. It was found that the medical personnel were not being treated equally in terms of transfers. A reasonably high degree of dissatisfaction was found among the doctors in terms of their remuneration. Only 11.25% doctors got promoted within 5 to 10 years of their joining the state services.

Regarding the perception on availability of work space, instruments, drugs, supporting staff, logistics and community support, the specialists were found to be satisfied but in case of generalists, only 64% are satisfied.

The survey showed that most of the infrastructural facilities including essential equipments were available in all the district hospitals and community health centres, barring a few in the latter. However, in the primary health centres, such facilities were not sufficiently available.

The results indicate that posting, perception regarding specialization, hierarchical satisfaction, quality of facilities and services and human resource policy are the determinants for the workforce availability.

## Discussions

The results of the study call for a need for better planning and management of human resource policy. Such planning should ensure transparency in deployment, transfers and promotions of doctors in the field. Increased and improved facilities should be made available to doctors in peripheral health institutions. Specifically, this would call for placing sufficient nursing staff, making laboratory technicians available, equipping with sufficient material like syringes, needles, cold chain equipments and surgical material.

## Competing interests

None declared

## Funding statement

The study was funded by the National Health Systems Resource Centre, New Delhi.

